# Damage before destruction? X-ray-induced changes in single-pulse serial femtosecond crystallography

**DOI:** 10.1107/S2052252525002660

**Published:** 2025-04-14

**Authors:** Lewis J. Williams, Amy J. Thompson, Philipp Dijkstal, Martin Appleby, Greta Assmann, Florian S. N. Dworkowski, Nicole Hiller, Chia-Ying Huang, Tom Mason, Samuel Perrett, Eduard Prat, Didier Voulot, Bill Pedrini, John H. Beale, Michael A. Hough, Jonathan A. R. Worrall, Robin L. Owen

**Affiliations:** a Diamond Light Source Harwell Science and Innovation Campus DidcotOX11 ODE United Kingdom; b School of Life Sciences University of Essex Wivenhoe Park ColchesterCO4 3SQ United Kingdom; c SwissFEL Paul Scherrer Institute 5232Villigen PSI Switzerland; d Department of Life Sciences Imperial College London LondonSW7 2AZ United Kingdom; e Research Complex at Harwell Harwell Science and Innovation Campus DidcotOX11 ODE United Kingdom; European Molecular Biology Laboratory, France

**Keywords:** XFELs, radiation damage, pulse duration, serial femtosecond crystallography

## Abstract

Serial femtosecond crystallography (SFX) exploits extremely brief X-ray free-electron laser pulses to obtain diffraction data before destruction of the crystal. However, during the pulse X-ray-induced site-specific radiation damage can occur, leading to electronic state and/or structural changes. Here, we present a systematic exploration of the effect of single-pulse duration and energy (and consequently different dose rates) on site-specific radiation damage under typical SFX room-temperature experimental conditions. For the first time in SFX we directly measured the photon pulse duration, varying from less than 10 fs to more than 50 fs, and used three pulse energies to probe in-pulse damage in two radiation-sensitive proteins: the iron-heme peroxidase DtpAa and the disulfide-rich thaumatin. While difference-map features arising from radiation damage are observed, they do not lead to significant change in refined atomic coordinates or key bond lengths. Our work thus provides experimental verification that average atomic coordinates are not significantly perturbed by radiation damage in typical SFX experiments.

## Abstract 2

Varied pulse-duration and pulse-intensity serial femtosecond crystallography data do not show significant signs of radiation damage under typical experimental conditions.

## 1. Introduction

Serial femtosecond crystallography (SFX) using X-ray free-electron lasers (XFELs) has opened a new frontier in structural biology. The short, femtosecond, duration of XFEL pulses allows time-resolved studies of fast nonreversible processes in challenging systems, exploiting the premise of diffraction before destruction (Orville, 2020 ▸; Barends *et al.*, 2022 ▸; Caramello & Royant, 2024 ▸). The results delivered by, and the promise of, SFX has led to the worldwide development of serial synchrotron crystallography (SSX), which utilizes many of the same sample-delivery techniques to probe slower processes (Pearson & Mehrabi, 2020 ▸). Beyond the possibility of probing extremely fast dynamics, SFX has an additional key advantage over SSX in that the brief durations (typically 10–50 fs) of the XFEL pulses can allow structures to be obtained that are essentially free of the site-specific artefacts of radiation damage. Such damage-free data collection is of particular importance for proteins containing high-valence-state metals or other redox centres, where the electronic state of the metal centre is often perturbed even in very low X-ray dose synchrotron experiments (Pfanzagl *et al.*, 2020 ▸; Ebrahim *et al.*, 2019 ▸; Moody & Raven, 2018 ▸). A key question in SFX experiments is: when and under what conditions do these fast damage processes modify the electron density and the derived atomic coordinates that are the primary result of the diffraction experiment? In this study, we quantify the XFEL pulse parameters (duration and intensity) for which this ‘damage-free’ premise may be considered to be valid in single-pulse SFX experiments under relatively typical experimental conditions for pulse energy, X-ray energy and beam size. Previous work has shown that for short XFEL pulses at modest flux densities, metalloprotein structures differ from those obtained using synchrotron radiation and are in agreement with quantum-mechanical/molecular-mechanics (QM/MM) simulations of the undamaged protein (Tosha *et al.*, 2017 ▸). This leads to a good level of confidence that such structures may be considered to be free of the effects of radiation-induced chemistry. A key question that we aim to address is whether the use of longer pulses, that may be less challenging to generate, are deleterious for SFX experiments and if a drive for shorter pulse durations for standard SFX experiments is warranted.

Key to assessment of the effect of pulse parameters on beam-induced changes is accurate quantification of the pulse duration and intensity. Accurate measurements of XFEL pulse profile and duration can be obtained from the imprint of the lasing process on the electron beam. This can be achieved by streaking the electron beam after the undulator, either with radiofrequency systems (Behrens *et al.*, 2014 ▸) or using a wakefield structure as performed here (Dijkstal *et al.*, 2022 ▸). In the absence of such direct measurements, the pulse duration can only be inferred through indirect methods, for example from the electron-bunch length or the FEL spectra. In this work, we used a wakefield structure to directly measure the pulse duration in SFX experiments for the first time.

XFEL pulses cause high X-ray doses to be absorbed by crystals in an extremely short period of time. Provided that the pulse is sufficiently brief, the rapid deposition of absorbed energy results in crystal destruction via Coulomb explosion (Neutze *et al.*, 2000 ▸) or hydrodynamic expansion (Ditmire, 2016 ▸) at some time after the diffracted photons leave the crystal. The use of short pulses does not guarantee that a SFX structure is damage-free, however: before destruction and during diffraction, X-ray-induced electronic state changes occur with subsequent atomic motions and rearrangement, and indeed site-specific damage was inferred in early XFEL experiments using pulses varying in nominal duration from 70 to 400 fs (Lomb *et al.*, 2011 ▸). Before crystal destruction, site-specific damage may be reflected by a deterioration in global data-quality metrics: the absorption of X-ray photons, and resulting atomic ionizations, at various locations in different unit cells results in a loss of long-range order with a concomitant loss of high-resolution reflections (Lomb *et al.*, 2011 ▸; Chapman *et al.*, 2011 ▸). This loss of order means that accumulated Bragg intensity is no longer proportional to the incident pulse intensity for longer pulses; this ‘Bragg termination’ was shown to result in loss of high-resolution (higher than 6.6 Å) reflections after ∼30 fs when using soft (2 keV) X-rays (Barty *et al.*, 2012 ▸).

More recent experimental work to understand damage processes used deliberately highly damaging experimental conditions to produce and characterize site-specific radiation damage in SFX structures. Nass, Schlichting and coworkers observed radiation damage including atomic displacements in the electron-rich [4Fe–4S] clusters of ferredoxin using very high pulse energies, close in energy to the Fe *K* edge, pulse durations of 80 fs and a nanofocused beam (Nass *et al.*, 2015 ▸). This work was followed by atomistic simulations which indicated that photo-ionization followed by Auger decay would lead to significant displacement of both Fe and S atoms within a 20 fs XFEL pulse, with motion predicted to be larger for the lighter S atoms (Hau-Riege & Bennion, 2015 ▸).

The influence of atomic mass on susceptibility to beam-induced change was also highlighted by simulations of photosystem II (PSII) exposed to pulses between 10 and 50 fs in duration, which suggested that lighter atoms such as oxygen would be displaced more significantly than the manganese ions in the active site, with the magnitude of displacement increasing as a function of pulse duration (Amin *et al.*, 2016 ▸). Further simulations predicted displacements of 0.25 and 0.39 Å of Mn and O atoms, respectively, within a 50 fs pulse, with displacement dependent on both interatomic bonds and pulse intensity (Amin *et al.*, 2017 ▸). Comparison of PSII structures collected to a resolution of 2.4 Å with contrasting pulse intensities and durations at SACLA (0.3 mJ, 7 fs pulses) and LCLS (2 mJ, 35 fs pulses) showed no differences in active-site geometry, however, with this likely to be due to the much lower pulse intensities used compared with those in the simulation (Ibrahim *et al.*, 2020 ▸).

The susceptibility of heavier atoms to XFEL-induced site-specific damage extends beyond metals to sulfur sites, such as disulfide bonds, with an expectation that S atoms will lose six electrons after 30 fs with a resultant elongation of disulfide bonds (Caleman *et al.*, 2020 ▸). These simulations agreed with the experimental observations of Nass *et al.* (2020 ▸), who applied a two-pulse, two-colour approach to thaumatin and lysozyme crystals, making use of two 15 fs XFEL pulses separated by time delays of several tens of femtoseconds (Nass *et al.*, 2020 ▸). This approach enabled site-specific radiation damage induced by the pump pulse (above the iron edge) to be characterized by the probe pulse (below the iron edge). Clear trends of radiation-induced changes at metal sites and disulfide bonds were observed with time delays as short as 18 fs, resulting in movements in atomic positions of several ångströms. Notably, the magnitude of the elongation of disulfide bonds was proportional to the delay time between the X-ray pump and X-ray probe pulses. More unexpectedly, distinct changes were also observed in the protein backbone close to carbonyl oxygens in β-sheet regions and at aromatic side chains.

How XFEL induced damage evolves over timescales longer than the pulse is a key consideration for approaches such as multi-hit SFX (Holmes *et al.*, 2022 ▸) and X-ray pump–probe (XRPP; Bolton *et al.*, 2024 ▸). In the former, an absence of observable beam-induced damage between exposure to two successive pulses of the EuXFEL separated by <1 µs raises the possibility of using such a megahertz source to probe sub­microsecond structural dynamics. Each pulse resulted in a dose of ∼100 kGy, with lysozyme structures from the first and second pulses showing no X-ray-induced change. This may not be the case for more radiation-sensitive samples such as metalloproteins: the XRPP approach used an attenuated SACLA pump pulse to trigger a transition between iron redox states which were then probed by a second unattenuated pulse 33 ms later, revealing structural change to a key active-site residue.

Key practical questions for single-pulse SFX experiments include (i) what are the maximum ‘safe’ pulse durations and intensities for determining unaltered structures and (ii) when these criteria are not satisfied, what are the fingerprints of XFEL-induced damage and where are they most likely to be observed? The answer to this question may depend on a convolution of these two parameters, *i.e.* in principle certain pulse intensities could be ‘safe’ at shorter but not at longer pulse lengths. For this reason, we have systematically varied both of these parameters to also assess their interdependence. These questions are especially pertinent for radiation-sensitive proteins such as heme proteins in high-valence oxidation states or those containing disulfide bonds, but also for protein crystals that might typically be considered relatively radiation-hard.

The radiation-sensitive dye-type heme peroxidase DtpAa possesses, in the ferric [Fe(III)] state, a water molecule bound to the catalytic iron heme at the distal face. The distance between this water and the heme iron has previously been shown to be extremely radiation-sensitive; the water dis­associates and moves away from the iron with accumulated dose in SSX experiments as a result of iron photoreduction, followed by downstream structural rearrangements (Ebrahim *et al.*, 2019 ▸). We have previously obtained the ground-state structure of this protein using 10 fs XFEL pulses at SACLA (Ebrahim *et al.*, 2019 ▸). In contrast, thaumatin contains no heavy atoms but has eight disulfide bridges that provide an alternative metric for radiation damage and has been used in previous radiation-damage studies including those by Nass *et al.* (2020 ▸)*.*

In this work, we have explored the effect of different pulse durations (7.9–53 fs) and pulse energies (10–100 µJ) on the SFX structures of DtpAa and thaumatin: this variation of beam and sample parameters led to a significant range of dose rates being explored. Crucially, other sample-delivery and beamline parameters were kept identical, allowing us to exclude differences between beamlines and sample batches. Data were collected to high resolution (>1.55 Å), allowing us to resolve any subtle structural changes and to identify any lower occupancy structures resulting from radiation damage. The high-resolution diffraction data obtained yielded dose-dependent difference map features consistent with ionization but, intriguingly, under the typical SFX experimental conditions that were used, data from both highly radiation-sensitive proteins did not reveal any significant or systematic change in the atomic coordinates that are the outcome of the crystallo­graphic experiment. We observe that under the pulse parameters used, even radiation-sensitive metalloprotein active-site structures are for practical purposes relatively insensitive to pulse length and intensity.

## 2. Materials and methods

### 2.1. XFEL setup and SFX data collection

Data were collected over a 30 h beamtime in September 2023 at the Cristallina experimental station of the SwissFEL ARAMIS beamline using the SwissMX endstation (Prat *et al.*, 2020 ▸). To shield the detector from the excess X-ray scattering of the in-air beam path, pre-sample and post-sample scatter guards surround the beam from the exit of the on-axis viewing (OAV) system to the detector face, leaving a gap of approximately 15 mm at the sample-interaction point. Diffraction data were recorded using an 8 megapixel JUNGFRAU integrating detector (Mozzanica *et al.*, 2016 ▸) at a distance from the sample of 111 mm.

SwissFEL was in 100 Hz mode with each self-amplified spontaneous emission (SASE) pulse giving a central photon energy of approximately 12.03 keV. Pulse intensities were measured first at a gas monitor directly after the diagnostic wakefield structure and then at the sample position using a JUNGFRAU 1.5M detector (see below and Supplementary Fig. S2).

The X-ray beam was focused using Kirkpatrick–Baez (KB) mirrors to 3.8 × 2.1 µm (full-width half-maximum; FWHM) at the sample position using the 24 fs pulse duration and this beam size was maintained for all other measurements. The beam profile was measured by scanning a tungsten blade through the X-ray beam. Changing pulse duration resulted in a change of pulse energy and a differing response of the gas monitor used to record the pulse intensity. This necessitated recalibration of the pulse energy for each of the durations. For this experiment, four pulse durations could be delivered from the machine, 7.9, 23.8, 41.3 and 52.7 fs (FWHM), using the setup described in the supporting information.

**Figure 1:**
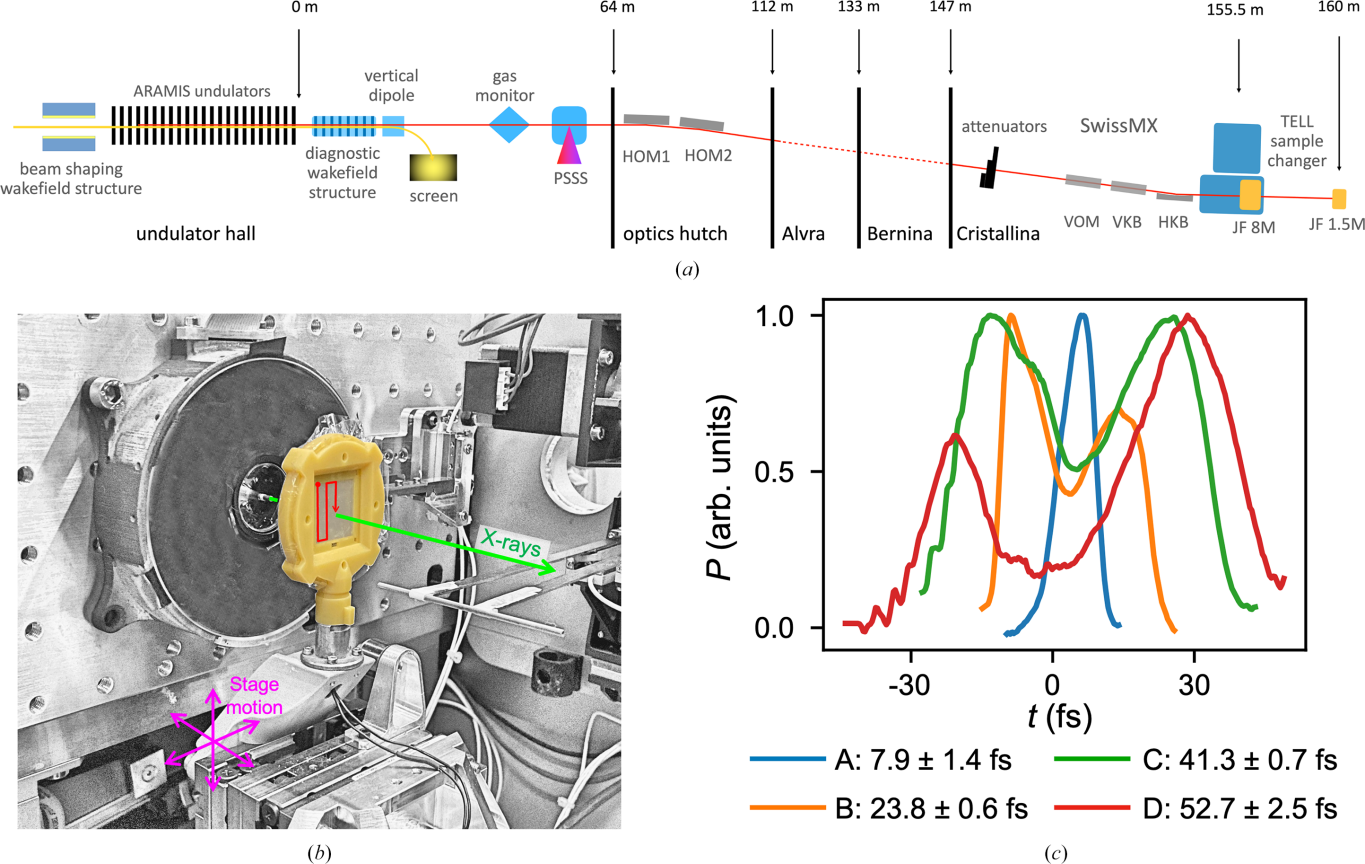
Overview of the experimental setup employed at SwissFEL during the experiment and measurements of the pulse durations used. (*a*) An overview of the optics and devices in the Cristallina beamline path with approximate distances from the end of the undulator line. The acronyms used are as follows: PSSS, photon single-shot spectrometer; HOM, horizontal offset mirror; VOM, vertical offset mirror; HKB/VKB, horizontal and vertical focusing mirrors; JF, Jungfrau. (*b*) Experimental setup used, highlighting the X-ray beam path, polymer fixed target (yellow) and chip motion. (*c*) Temporal profiles of XFEL pulses, centred on *t* = 0, at different machine configurations A–D, measured with the diagnostic wakefield structure averaged from 20 single-shot measurements.

After each pulse duration had been set for the machine, the total mean number of photons per pulse was measured directly with a 1.5 megapixel JUNGFRAU detector 4.84 m downstream of the sample position [Fig. 1 ▸(*a*)]. The transmission of the beam from the gas monitor to the detector was calculated using *XOP* (Sánchez del Río & Dejus, 2011 ▸), taking into account the different beamline elements and air path [Supplementary Fig. S2(*a*)]. A 2.8 mm cover was put on the face of the 1.5 megapixel JUNGFRAU detector to provide sufficient attenuation to measure the direct beam [Supplementary Fig. S2(*b*)]. The mean pulse energy at multiple filter transmissions ranging from 1.000 to 0.005 was directly measured on the covered detector. These data were used to back-calculate standard curves so that consistent pulse energies of 10, 50 and 100 µJ were applied at the sample position for all pulse durations [Supplementary Fig. S2(*c*)]. 100 µJ was the highest common pulse energy that could be obtained for all pulse durations and corresponds to 5.3 × 10^10^ photons per pulse. Care was taken to minimize any differences between experimental parameters other than the pulse duration or pulse energy for each experiment.

### 2.2. DtpAa protein expression and purification

The DtpAa-pET-28a plasmid (carrying the Y389F mutation) was transformed into *Escherichia coli* C43(DE3) cells and 10 ml precultures were grown overnight [low-salt LB medium (Melford), 50 µg ml^−1^ kanamycin]. These precultures were used to inoculate 1.4 l cultures [low-salt LB medium (Melford), with additional 5 g l^−1^ NaCl, 50 µg ml^−1^ kanamycin] grown at 37°C and 180 rev min^−1^ until the OD_600_ reached 0.8–1.0, at which point expression was induced by the addition of isopropyl β-d-1-thiogalactopyranoside (final concentration 500 µ*M*). Concurrent with induction, cultures were supplemented with δ-aminolevulinic acid (final concentration 500 µ*M*) and iron citrate (final concentration 100 µ*M*), and CO gas was bubbled into the cultures for ∼30 s. The flasks were sealed with rubber bungs and incubation of the cultures continued at 30°C and 100 rev min^−1^ for a further 16–20 h. The cells were centrifuged (3990*g*, 20 min, 4°C), the supernatant was decanted and the pellets were resuspended in buffer *A* (50 m*M* Tris–HCl, 500 m*M* NaCl, 20 m*M* imidazole pH 7.5). The cells were lysed using an EmulsiFlex-C5 cell disrupter (Avestin) and centrifuged (39 190*g*, 45 min, 4°C). The supernatant was loaded onto a HisTrap HP 5 ml (Cytiva) column equilibrated with buffer *A*. The column was washed with 5–10 column volumes of buffer *A* and the protein was eluted with buffer *B* (50 m*M* Tris–HCl, 500 m*M* NaCl, 500 m*M* imidazole pH 7.5) using a 45 min linear concentration gradient. The DtpAa-Y389F variant-containing fractions were then combined and concentrated to 2 ml using a 10 kDa Ultraspin concentrator (Vivaspin) and loaded onto a Superdex 200 16/600 size-exclusion column (GE Healthcare) equilibrated with buffer *C* (20 m*M* sodium phosphate, 150 m*M* NaCl pH 7.0). The DtpAa-Y389F-containing fractions were combined, concentrated to the desired concentration and stored at 4°C. The protein concentration was calculated via UV–Vis spectroscopy (Cary 60 UV–Vis spectrophotometer) using ɛ_280_ = 46 075 *M*^−1^ cm^−1^.

### 2.3. DtpAa crystallization

Batches of microcrystals were set up in 1.5 ml Eppendorf tubes using a 1:3(*v*:*v*) ratio of 10 mg ml^−1^ DtpAa in buffer *C* and a mother-liquor solution consisting of 12%(*v*/*v*) PEG 3350, 100 m*M* HEPES pH 7.0. The total solution volume was 400 µl. Batches were set up within 48 h of protein purification. Crystals of around 30 µm in size grew over 24–48 h at 18°C.

### 2.4. Thaumatin crystallization

100 mg of thaumatin (Sigma, catalogue No. T7638) was dissolved in 1 ml Milli-Q water, reaching a concentration of 100 mg ml^−1^; solutions were vortexed to ensure complete dissolution. Subsequently, 200 µl of the thaumatin solution was mixed with 200 µl 1.6 *M* sodium potassium tartrate crystallization solution. A tube of the resulting mixture was placed on a rotator operating at 20°C. After an incubation period of 16–18 h, thaumatin crystals grew to a size of 30–40 µm with high density. Crystals were stored at 4°C until sample loading and beamtime.

### 2.5. Data collection

For data collection, samples were loaded onto polymer fixed targets (Carrillo *et al.*, 2023 ▸) within a humidity stream. Loaded fixed targets were stored within a humidity enclosure with a typical time between fixed-target loading and data collection of 15 min. A spacing of 120 µm (horizontal and vertical) was used, with data collected from 25 000 positions per fixed target. Data collection took 250 s per fixed target, corresponding to a throughput of 4–5 chips per hour including chip exchange and alignment. Each crystal was exposed to a single XFEL pulse of varying duration and intensity as summarized in Supplementary Table S3. All data were measured at 21°C.

The SFX data were indexed, integrated and scaled using *CrystFEL* v.10.2 (White *et al.*, 2012 ▸); Bragg peaks were identified using the *peakfinder*8 algorithm and indexed using *XGANDALF* (Gevorkov *et al.*, 2019 ▸). Data were integrated using the flag rings-grad. Scaling and merging were performed using the unity model within the *CrystFEL* program *partialator*, and intense peaks were excluded with a max-adu of 10 000. This was performed in order to avoid artefacts arising from detector saturation, the level of which was assessed using the *peakogram-stream* tool within *CrystFEL* (Supplementary Fig. S4). Data were phased using Protein Data Bank (PDB) entries 6i43 (Ebrahim *et al.*, 2019 ▸; DtpAa) and 4axr (Cipriani *et al.*, 2012 ▸; thaumatin). The data-resolution cutoff was defined as the point at which the correlation coefficient decreased smoothly to 0.3. Structures were subsequently refined and rebuilt using alternate cycles of *REFMAC* (Murshudov *et al.*, 2011 ▸) or *Phenix* (Liebschner *et al.*, 2019 ▸) and then *Coot* (Emsley *et al.*, 2010 ▸), and validated using the PDB validation server. Electron-density figures were prepared using *PyMOL* (DeLano, 2002 ▸). Selected fully refined models were deposited in the PDB as indicated in Supplementary Tables S1 and S2. Errors in specific bond lengths were estimated via the diffraction precision indicator (DPI). Previously, use of the DPI to estimate coordinate error was benchmarked against the gold-standard full-matrix inversion method, with good agreement having been demonstrated (Cruickshank, 1999 ▸). A DPI value for each structure is calculated representing the uncertainty in position for atoms with the mean refined *B* factor of that structure. Subsequently, each atom in the refined structure is assigned an individual value derived from the overall DPI and the *B* factor of the specific atom using the approach of Gurusaran and coworkers as implemented in the *Online_DPI* server (Kumar *et al.*, 2015 ▸; Gurusaran *et al.*, 2014 ▸). The uncertainty estimates for each of the two atoms forming a bond can then be used to derive an estimate of the bond-length uncertainty as described by Helliwell (2023 ▸).

Isomorphous difference density maps were obtained using *Radiation-Induced Density Loss* (*RIDL*; Bury & Garman, 2018 ▸). *RIDL* calculates per-atom metrics to quantify electron-density changes between complete data sets. In calculating difference maps, *RIDL* uses phases from a refined model of a reference-dose data set (*n* in this example) and structure factors from later data sets *m* to produce *F*_o*n*_ − *F*_o*m*_ difference maps. In Figs. 3 and 4, the phases and reference model were provided by the data set on the *x* axis of the plot, *i.e.* all plots in the leftmost column use the phases of the 4 kGy data set. Difference maps were coloured so that red and green indicate loss and gain, respectively, of electron density in later data sets.

*RIDL* maps generated from data sets without a consistent resolution cutoff exhibited strong ripple features which may be assigned to Fourier truncation effects (Supplementary Fig. S7). These were particularly evident around heavier atoms such as iron. When consistent global resolution cutoffs were applied, the ripples were significantly reduced but still dominated around the heme iron. To thoroughly explore Fourier effects and minimize artefacts in the radiation-damage specific maps, we truncated data sets to consistent high-resolution cutoffs of 1.6, 1.75 and 2.0 Å, with the results leading us to utilize a resolution cutoff of 1.75 Å for difference-map generation. We note here that these density features occur only within the *RIDL* maps and not in the conventional electron-density and difference maps used in model building and refinement.

Scaling and refinement statistics for all samples are given in Supplementary Tables S1 and S2. Doses were calculated using *RADDOSE-XFEL* (Dickerson *et al.*, 2020 ▸) and are given in Supplementary Table S3. Doses reported here are the average dose in the exposed region (ADER), which takes into account the duration of the pulse length when calculating the dose absorbed by a crystal during the pulse.

## 3. Results

### 3.1. Generation of variable pulse durations

SwissFEL was used in 100 Hz mode, with each self-amplified spontaneous emission (SASE) pulse giving a central photon energy of 12.03 keV. The photon pulse duration of the FEL is approximately equal to the electron-bunch duration with sufficient quality to lase. The longest pulse duration was around 50 fs and was limited by the fixed bunch charge of 200 pC and the minimum peak current to achieve a good FEL performance. We produced shorter pulses first by compressing the electron bunch. This was limited to around 20 fs due to loss of beam quality and stability. Shorter pulses down to about 10 fs were produced by streaking the electron beam with passive wakefield structures installed upstream of the undulator. In a streaked electron beam, only a fraction of the electron beam is well aligned in the undulator and able to produce XFEL radiation. To generate the shortest possible pulses (<20 fs), an upstream wakefield was used to induce a transverse deflection of the beam and thus temporally shape pulses (Emma & Huang, 2004 ▸; Lutman *et al.*, 2016 ▸).

The pulse duration at each machine configuration was measured using a diagnostic wakefield structure (Dijkstal *et al.*, 2022 ▸) installed downstream of the undulator [Fig. 1 ▸(*a*)] and data were collected. The wakefield structure comprises two opposing plates with corrugated surfaces and allows the FEL pulse to be passively streaked and thus the temporal profile of the pulse to be reconstructed. Such explicit measurement of pulse duration and temporal profile is not typically undertaken in SFX experiments, with a simple numerical value usually being stated instead. Four pulse durations were delivered by the machine, 7.9 ± 1.4, 23.8 ± 0.6, 41.3 ± 0.7 and 52.7 ± 2.5 fs [Fig. 1 ▸(*c*)], and data were collected at the Cristallina experimental station of the SwissFEL ARAMIS beamline using the SwissMX endstation and polymer fixed targets [Fig. 1 ▸(*b*)].

### 3.2. Data-quality description

High-resolution SFX data sets were obtained for different combinations of pulse duration and pulse energy. Data sets varied in resolution between 1.44 Å (7.9 fs, 10 µJ data set) and 1.21 Å (41.3 fs, 100 µJ) for DtpAa and between 1.55 Å (7.9 fs, 10 µJ) and 1.38 Å (23.8 fs, 100 µJ) for thaumatin. These high-resolution data provide confidence in the potential to resolve and interpret subtle structural changes that may be caused by the XFEL pulse. No fewer than 6200 indexed lattices were used to form each data set. Data-scaling and refinement statistics are given in Supplementary Tables S1 and S2 and are summarized in Supplementary Fig. S3. There is no systematic trend in data-scaling metrics as a function of pulse duration or intensity. Wilson plots showed the expected linear decay of the log of diffraction intensity with (sinθ/λ)^2^ (Supplementary Fig. S3). Detector saturation was evident, particularly with the highest pulse energy (Supplementary Fig. S4), although for consistency detector saturation was taken into account when processing all data sets (see Section 2). No clear trend was observed between pulse duration and data-set quality, although the resolution cutoff was generally higher for 50 and 100 µJ data sets (Supplementary Fig. S5). Any systematic dependence of CC_1/2_ on pulse duration significantly reduced if data sets consisting of identical numbers of indexed images were compared. Two data sets (DtpAa 53 fs, 50 µJ; thaumatin 7.9 fs, 100 µJ) showed poor merging statistics (overall CC_1/2_ < 0.85), and this seems to be partly correlated to the number of indexed lattices, but these are nonetheless included for completeness.

### 3.3. Radiation damage in SFX structures of DtpAa

**Figure 2:**
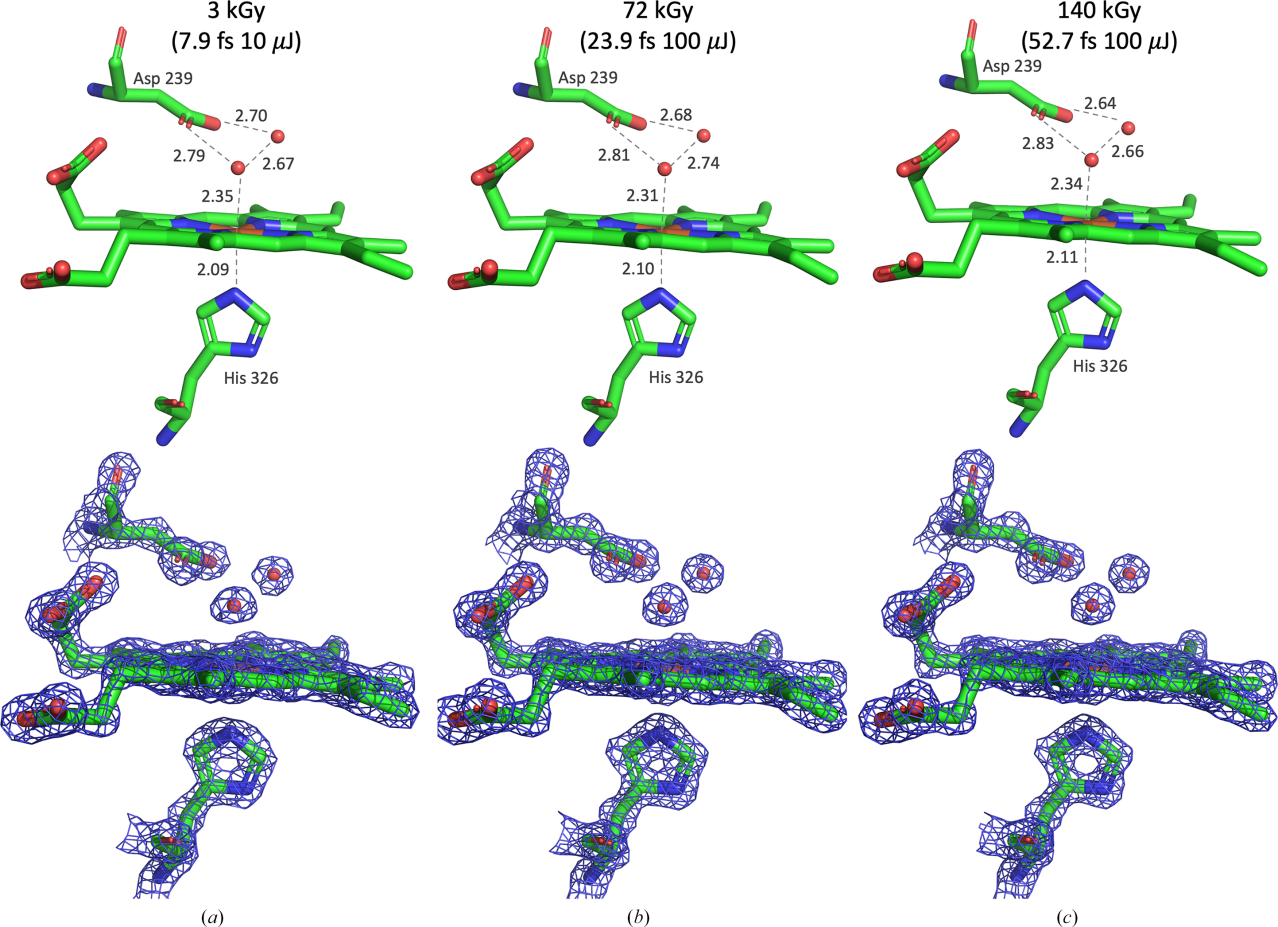
Heme-site structures showing interatomic distances (top row) and 2*F*_o_ − *F*_c_ electron density (bottom row) in DtpAa (chain *A*). Distances and density are shown for 7.9 fs, 10 µJ (*a*), 24 fs, 10 µJ (*b*) and 53 fs, 100 µJ (*c*) data sets. Doses given are the average dose in the exposed region of the crystal (ADER). All distances are in ångströms; electron density is contoured at 2σ.

A ground-state structure of DtpAa from SwissFEL was obtained using 7.9 fs, 10 µJ pulse data to 1.44 Å resolution to provide a reference point against which to compare subsequent structures, with the heme Fe(III)–H_2_O bond used as a primary indicator of damage. The electron density around the heme pocket was highly similar to that observed in our previous DtpAa SFX structure determined at SACLA (Ebrahim *et al.*, 2019 ▸). The Fe(III)–H_2_O bond distance of 2.40 ± 0.13 Å obtained at SACLA (10 fs pulses) was slightly elongated compared with the distance of 2.35 ± 0.10 Å observed at SwissFEL (7.9 fs pulses). Fig. 2 ▸ compares the 7.9 fs, 10 µJ SwissFEL structure with refined models obtained using longer pulse durations of 24 and 53 fs at high (100 µJ) intensity at SwissFEL. The electron density observed is extremely similar in all cases with no loss of H_2_O, side-chain or heme density. The Fe(III)–N^δ^–His bond is unchanged between structures. A slight decrease in the Fe(III)–H_2_O bond length to 2.31 ± 0.05 Å (24 fs) and 2.34 ± 0.06 Å (53 fs) was observed, although we note that these differences are comparable to the estimated error in the bond lengths and no systematic variation in bond length is observed (Supplementary Fig. S6).

No significant changes could be readily detected in the atomic coordinates of the refined DtpAa structures between data sets, and difference-map features were not sufficient to justify the modelling of any partial occupancy alternative structures consistent with a damaged state. In a previous serial synchrotron crystallography study, we identified an elongation of a heme Fe(III)–H_2_O bond to be a marker of radiation damage (Ebrahim *et al.*, 2019 ▸). DtpAa is a homodimer, and in chain *A* this bond length is the same within experimental error (Supplementary Table S4), as is the Fe(III)–N^δ^–His bond on the proximal side of the heme. This lack of variation outside experimental error includes the 24 fs, 10 µJ structure, which shows a slightly shortened Fe(III)–H_2_O bond length in Fig. 2 ▸(*b*). In chain *B*, the coordinating water is seen in two conformations (refined as 0.5 occupancy), as also noted in a previous SFX structure of DtpAa (Lučić *et al.*, 2021 ▸). The average *B* factors of both protein chains and the hemes also show no dependence on radiation dose (Supplementary Table S4). *RIDL* difference maps (Bury & Garman, 2018 ▸) between pairs of data sets were calculated, as described in Section 2, to provide a sensitive probe of radiation damage. *RIDL* difference maps between pairs of DtpAa structures revealed significant Fourier ripple effects in the difference maps centred around the Fe(III) atom, which made the interpretation of difference density caused by radiation damage challenging (Supplementary Fig. S7). While *RIDL* maps showed some electron-density loss or change, both visual inspection of maps and refinement statistics show that this electron-density loss has no detrimental effect on the final models.

### 3.4. Radiation damage in SFX structures of thaumatin

**Figure 3:**
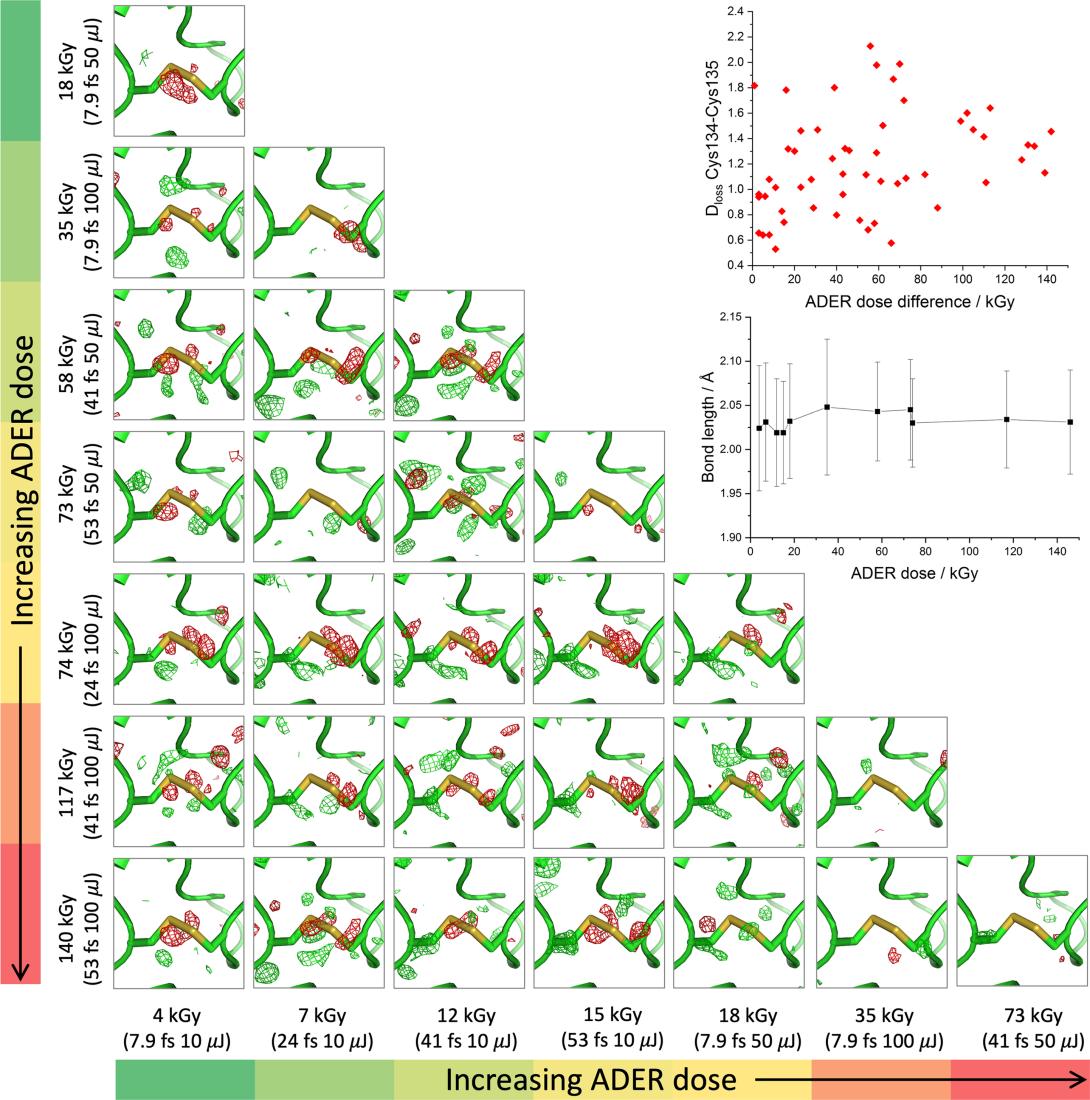
*RIDL* isomorphous difference maps comparing thaumatin data sets at the exemplar disulfide bond Cys134–Cys145. Rows and columns are sorted in order of average dose in the exposed region (ADER), with lowest doses at the top and left-hand sides. If site-specific damage is occurring and is a function of ADER, the largest features should appear towards the bottom left of the figure where the largest differences in dose occur. The top inset compares *D*_loss_ (in e Å^−3^) at Cys134–Cys145 between pairs of data sets, as calculated by *RIDL,* as a function of the difference in ADER dose between each data set. The lower inset shows the S—S bond length as a function of ADER dose; this is shown for all disulfide bonds in Supplementary Fig. S6. An analogous figure with columns and rows sorted by beam power rather than dose is given in Supplementary Fig. S8. All difference maps are contoured at 3σ.

**Figure 4:**
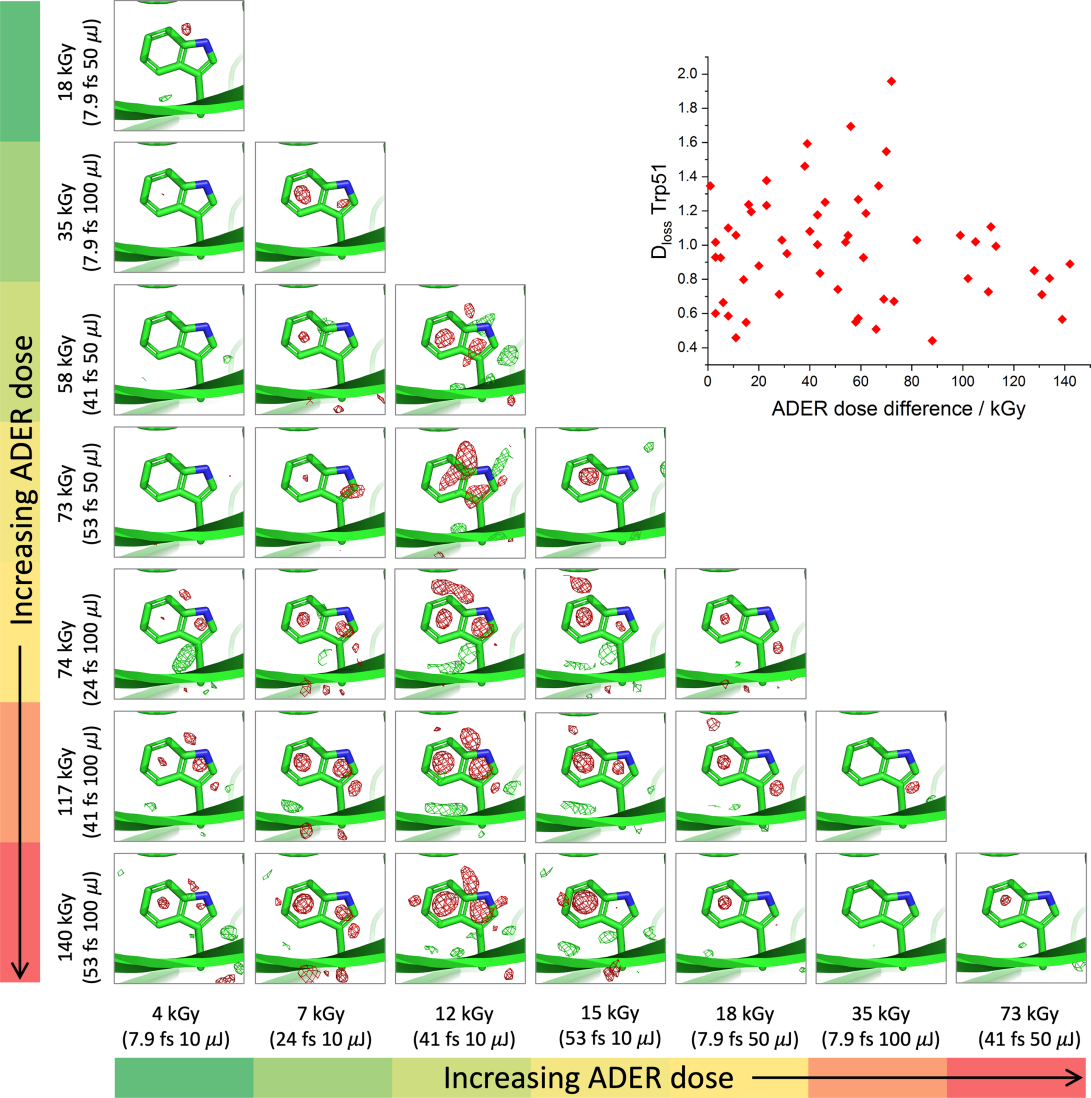
*RIDL* isomorphous difference maps comparing thaumatin data sets at Trp51. Rows are sorted in order of average dose in the exposed region (ADER), with lowest doses at the top. All difference maps are contoured at 3σ. The inset compares *D*_loss_ (e Å^−3^) at Trp51 between pairs of data sets as a function of the difference in ADER dose between each data set. An analogous figure with columns and rows sorted by beam power rather than dose is given in Supplementary Fig. S9.

All SFX structures of thaumatin were refined to completion (Supplementary Table S2). Difference density features were observed at all disulfide bonds. In *RIDL* maps, clear features were also visible at carbonyl oxygens in β-sheet regions, and many aromatic residues appear to be partly ‘hollowed out’ with difference-map features in the centre of the aromatic rings. Figs. 3 ▸ and 4 ▸ show trends in *RIDL* difference density at the exemplar disulfide bond Cys134–Cys145 and at the residue Trp51 between pairs of data sets. Fig. 3 ▸ shows disulfide-bond difference density features for pairwise comparisons between data sets ordered by the average dose in the exposed region of the crystal (ADER). Data-set pairs with the largest difference in dose are towards the bottom left. It is evident that a weak trend exists where data-set pairs with larger differences in dose seem to be associated with larger difference peaks at the disulfide bond itself. We note that differences are not reflected in 2*F*_o_ − *F*_c_ maps and that the difference-map features are not sufficient to model modified positions for the S atoms or to change their occupancy. A similar plot showing the difference density around the aromatic Trp 51 residue is shown in Fig. 4 ▸. Here strong difference-map features are largely confined to the centre of the aromatic rings, consistent with ionization effects causing a loss of electrons from the ring system and, while a similar trend is observed, differences between one data set (41 fs, 10 µJ) and others are the primary feature, emphasizing the small magnitude of differences elsewhere. Supplementary Figs. S8 and S9 show Figs. 3 ▸ and 4 ▸ replotted with data sets sorted by incident beam power (*i.e.* increasing dose rate) rather than increasing dose, with the insets showing exemplar 2*F*_o_ − *F*_c_ density at each site at two extremes of dose.

**Figure 5:**
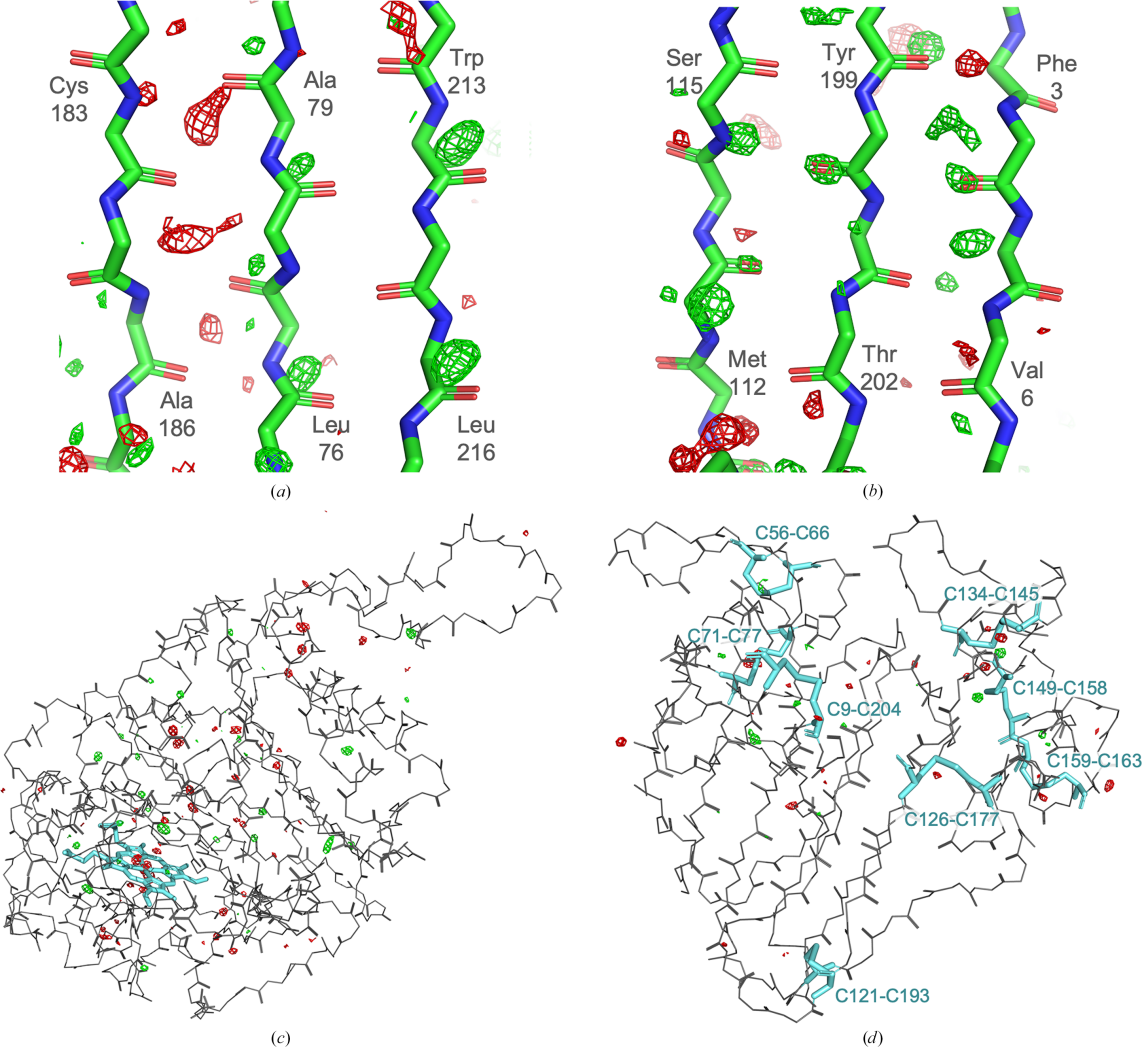
Global difference-map features in DtpAa and thaumatin. *RIDL* isomorphous difference maps comparing 7.9 fs, 10 µJ and 53 fs, 100 µJ data sets in β-sheet regions in DtpAa (*a*) and thaumatin (*b*) contoured at 3σ, highlighting protein-dependent differences. Peaks are observed in DtpAa to occur in the spacing between β-sheets and not necessarily where hydrogen bonds exist. Whole-molecule *RIDL* difference maps again comparing 7.9 fs, 10 µJ and 53 fs, 100 µJ data sets in DtpAa (*c*) and thaumatin (*d*) contoured at 4σ to highlight dominant features. Heme-group and disulfide bonds are highlighted in cyan as stick representations.

In the two-colour experiments by Nass *et al.* (2020 ▸), intriguing patterns of difference density were evident in *RIDL* maps around the hydrogen-bond networks making up the β-sheet secondary structure, particularly around carbonyl O atoms and backbone N atoms (the estimated ADER dose for the Nass pump pulse is 350 kGy). *RIDL* maps for β-sheet regions are shown in Figs. 5 ▸(*a*) and 5 ▸(*b*) for DtpAa and thaumatin between two data sets collected at extremes of dose (3 and 4 kGy at 7.9 fs, 10 µJ and 140 and 146 kGy at 53 fs, 100 µJ for DtpAa and thaumatin, respectively). Although we observe similar difference-map peaks at carbonyl O atoms in the thaumatin β-sheet these are not repeated in DtpAa, suggesting this could be an enzyme-dependent change. Figs. 5 ▸(*c*) and 5 ▸(*d*) highlight global differences between the 7.9 fs, 10 µJ and 53 fs, 100 µJ DtpAa and thaumatin data sets, with dominant peaks at the heme and disulfides, respectively. Plots of metrics such as *D*_loss_(atom), which quantifies electron-density loss near atoms between data sets, generated by *RIDL* at ‘top damage sites’ and at disulfide S atoms, showed no systematic change as a function of data set, with differences between data sets on the level of noise, as illustrated for the disulfide bond Cys134–Cys145 and Trp51 in the insets in Figs. 3 ▸ and 4 ▸, respectively.

Thaumatin data sets were fully refined in order to assess whether the observed features in difference or *RIDL* maps corresponded to a change in the end product of the experiment, *i.e.* the set of coordinates and *B* factors deposited in the Protein Data Bank (wwPDB Consortium, 2019 ▸). In no cases were the *RIDL *map features sufficiently large to justify the modelling of a partial occupancy state different to the ground-state structure. In order to establish whether any differences for coordinates were significant, refined structures were subjected to DPI error estimation in both coordinate positions and S—S bond length, with no systematic trend being observed (shown in the inset in Fig. 3 ▸ for Cys134–Cys145 and in Supplementary Fig. S6 for all thaumatin disulfide bonds). In order to confirm that an absence of disulfide-bond elongation in thaumatin was not due to refinement restraints, refinement was repeated with all cysteines modelled as alanines and unbound S atoms in different conformations added to the model at the positions of the cysteine sulfurs. After refinement to completion of models modified in this way, no systematic increase in S—S distances was observed.

Notably, our data are of substantially higher resolution than those used for previous two-colour experiments on thaumatin (2.3 Å, which revealed substantial atomic movements), providing additional confidence that our study would have resolved even very subtle differences that may have occurred in models or in identifying low-occupancy damaged states. Unexpectedly, in contrast to the previous pump–probe experiments, where large and unambiguous movements in atomic positions were clearly evident, our single-pulse data indicate that there is no obvious damaged component even when using the longest pulses (see Section 4).

Overall, for both DtpAa and thaumatin the extent of difference density appears to correlate with the average dose in the exposed region (ADER) more closely than with pulse duration. Superposition of structures with the least- and most-damaged structures resulted in an all-atom root-mean-square deviation (r.m.s.d.) that is around half the estimated standard deviation (e.s.d.) value for the atomic coordinates, meaning that they are identical within experimental error. In other words, the refined coordinates deposited in the PDB would have been identical within experimental error regardless of difference-map features that may have been observed. Note that this is true even for the high resolutions of our structures, in which we might expect to be able to resolve any significant structural changes that had occurred.

## 4. Discussion

The directly measured temporal pulse profiles exhibit multiple maxima rather than a single Gaussian peak, with the full-width half-maximum derived from an r.m.s. analysis. Despite the presence of complex pulse profiles, our data represent real-world delivery of different pulse durations and we anticipate that other XFEL beamlines will utilize similar approaches in the future to directly characterize the temporal profile of X-ray pulses delivered to the sample position.

Our data reveal that when comparing pairs of data sets only relatively subtle electron-density changes in *RIDL* maps are observed. These differences do not appear to vary systematically as a function of the average dose in the exposed region (ADER), incident pulse duration or intensity. The small magnitude of these difference-map features meant that care had to be taken to avoid misinterpretation due to Fourier truncation ripples around heavy atoms, in particular S and Fe (see Section 2).

While there is no clear systematic dependence, the extent of the difference-map features appears to follow the ADER dose estimate more closely than differences in the pulse duration. While difference density features are observed, most clearly in *RIDL* maps, essentially no differences are present in 2*F*_o_ − *F*_c_ maps and the r.m.s.d. between refined structures is small and in fact is smaller than the experimental error in the average atomic coordinates. Significantly, the Fe(III)–H_2_O bond length in the structures of DtpAa did not show any of the elongation that is typical in millisecond-timescale SSX structures following X-ray-driven reduction of Fe(III) (Ebrahim *et al.*, 2019 ▸). The experimental parameters at SwissFEL may be compared with our previous structure of this enzyme obtained at SACLA (10 fs, nominal pulse energy 37.6 µJ, ADER 132 kGy). Similarly, in thaumatin we did not observe the movement of S atoms and the consequent elongation of disulfide bonds even in the refined 53 fs, 100 µJ model corresponding to the highest dose structure. Minor populations of damaged states that do not affect the average atomic coordinates may develop over the duration of pulses: refinement against extrapolated structure factors, which is beyond the scope of this report, might yield models representing these damaged states

When comparing site-specific radiation damage in SFX and synchrotron experiments it is essential to remember that the mechanisms of damage differ greatly, even though the many susceptible sites (*e.g.* metals, S atoms) may be similar. In typical synchrotron dose-series experiments taking place on millisecond to second timescales, the reduction of metals and disulfides is typically observed. In contrast, on the femtosecond timescale more direct ionization effects are observed upon the absorbing atoms and bonds that they make. Features in our electron-density maps are consistent with a loss of electrons, *i.e.* ionization around the disulfide bridges, within aromatic amino acids and at carbonyl oxygens in the main chain.

An intriguing result is that we observe very limited changes to atomic coordinates even at the highest pulse energies and the longest pulse duration. A key question is why the changes in difference maps do not correspond to coordinate changes. The relatively subtle difference-map features do not allow any low-occupancy structurally distinct damaged state to be modelled even at the high resolutions achieved in our work. These observations therefore indicate that the key outcome of the SFX experiment (atomic coordinates) is essentially identical regardless of how these parameters were varied across our study, and therefore in practical terms pulse duration and energy may not be a significant factor in outcomes of XFEL SFX under typical experimental conditions. The sulfur–sulfur bond length shown in Fig. 3 ▸, for example, can essentially be considered to be invariant across different experimental parameters, suggesting that for all pulse durations and intensities used it closely reflects the undamaged structure.

Several SFX radiation-damage studies have previously been conducted and it is instructive to compare the results from these with our data. The only previous SFX radiation-damage study conducted on an iron-containing system used the highly electron-rich [4Fe–4S] cluster of ferredoxin combined with an experimental design that aimed to maximize the chances of observing damage, specifically an 80 fs, 1.5 mJ pulse that was focused into a 200 × 200 nm beam size using radiation close to the Fe *K* edge (Nass *et al.*, 2015 ▸). This study revealed large changes to electron density around the 4Fe–4S cluster in comparison to previous synchrotron single-crystal experiments, consistent with a high level of radiation damage. In particular, S atoms in the [4Fe–4S] cluster were significantly displaced in the 80 fs data electron density. In addition, Tosha *et al.* (2017 ▸) determined an SFX structure (10 fs pulses) of cytochrome P450Nor and showed that this was structurally distinct from both low-dose and high-dose synchrotron-derived structures while corresponding very well to the QM/MM prediction of the undamaged state. The latter study provides good evidence that under these conditions with a 10 fs pulse duration the resulting structure may be considered to be undamaged.

In contrast, we wished to explore experimental regimes more representative of typical SFX data collection in order to establish safe operating conditions for working with highly radiation-sensitive samples. Our maximum pulse energy was some 15 times lower than that used in the ferredoxin study, with a larger beam size that further reduced the maximum energy density, which thus became three orders of magnitude lower. We note that despite this reduction even our maximum pulse energy of 100 µJ was sufficient to cause a high level of detector saturation (Supplementary Fig. S5). We also worked with an X-ray energy remote from the Fe edge and which permitted high-resolution data collection, allowing any subtle structural changes to be resolved. Under these conditions we did not observe any structural changes around the Fe atom as a result of pulse duration or pulse energy.

For thaumatin our results for longer single pulses may be compared with those from a ‘two-colour’ pump–probe study on the same protein in which a 15 fs pump pulse was followed after a 10–100 fs time delay by a second 15 fs pulse. The overall pattern of difference-map features bears some similarities and some differences between single-pulse and two-colour experiments. In the two-colour experiment an increase in the length of disulfide bonds was clearly evident, consistent with bond breakage. In this case, negative difference density features were observed directly between the two S atoms forming the bond, with positive difference density adjacent to the S atoms, both of which are consistent with changes to the bond and atomic positions. In our case, while there are more density features in the vicinity of the disulfide bridges these do not directly correspond to the position of the bond or individual atoms and so are harder to relate to specific structural changes. Differences observed between these two experiments might be attributable to our use of a single pulse. Diffraction intensities accumulate over the duration of the pulse and thus will represent a mixture of states. This effect may be exaggerated due to the bimodal distribution of photons over the pulse duration [Fig. 1 ▸(*c*)]. In the two-colour study the pulse energy density was higher than in the experiments reported here, albeit by a much smaller factor than was the case for the ferredoxin data. It is likely that the longer wavelength of the two-colour experiment may have contributed to a higher effective dose being absorbed by the crystals. The standard pulse durations of different XFEL sources/beamlines vary between 10 and 30 fs; often, these are chosen to maximize the stability and quality of the beam. Our data argue that under typical conditions pushing for very short pulse durations at the expense of other factors may not be required. Pulse intensities much greater than 100 µJ are available at other sources and damage effects may be apparent on these timescales if the pulse intensity is increased significantly beyond this. We note that under our experimental conditions high-resolution data were obtained even with the lowest pulse energy of 10 µJ.

In conclusion, we explored site-specific radiation damage resulting from XFEL pulses between 7.9 and 53 fs and between 10 and 100 µJ in single-pulse SFX data, representative of typical experimental conditions using current generation detectors. Two radiation-damage sensitive proteins containing heme-Fe and disulfide bridges were studied. Subtle difference-map features exist that increase with the ADER dose, but these are sufficiently small that modelling a population of damaged states cannot be justified and they do not lead to any significant difference in the refined atomic coordinates arising from the experiment. We thus demonstrate that under typical SFX data-collection conditions, the structure of these two radiation-sensitive proteins was not significantly perturbed by radiation-damage effects, although electron-density changes were indicated by *RIDL* difference density maps. Under these conditions the use of longer XFEL pulses may be considered reasonable and will still result in refined structures that represent the nonperturbed structural state. These data thus suggest that under the experimental conditions of typical current SFX data collection, site-specific radiation damage is unlikely to be a major concern. We emphasize that X-ray-induced changes are certainly occurring within the duration of the pulse, but our results show that, under the typical pulse parameters used, these are nonsystematic and thus are not visible as a change in the coordinates obtained by X-ray crystallography. Even in cases where multiple maxima were observed within the nominal duration of the pulse the refined coordinate changes were not significant. Currently, many SFX experiments can only use attenuated XFEL pulses because of the limited dynamic range of existing detectors. However, the development of improved detectors where the full intensity of current and future XFEL beamlines could be utilized in diffraction experiments could potentially lead to much more severe radiation-damage effects.

## 5. Related literature

The following references are cited in the supporting information for this article: Juranić *et al.* (2018 ▸) and Tiedtke *et al.* (2008 ▸).

## Supplementary Material

PDB reference: thaumatin, 7.9 fs, 10 µJ, 9epe

PDB reference: 7.9 fs, 50 µJ, 9eqr

PDB reference: 7.9 fs, 100 µJ, 9eqs

PDB reference: 23.8 fs, 10 µJ, 9eqt

PDB reference: 23.8 fs, 100 µJ, 9equ

PDB reference: 41.3 fs, 10 µJ, 9eqv

PDB reference: 41.3 fs, 50 µJ, 9eqx

PDB reference: 41.3 fs, 100 µJ, 9eqy

PDB reference: 52.7 fs, 10 µJ, 9eqz

PDB reference: 52.7 fs, 50 µJ, 9er0

PDB reference: 52.7 fs ,100 µJ, 9er1

PDB reference: DtpAa, 7.9 fs, 10 µJ, 9epd

PDB reference: 23.8 fs, 10 µJ, 9epg

PDB reference: 23.8 fs, 100 µJ, 9epk

PDB reference: 41.3 fs, 10 µJ, 9epj

PDB reference: 52.7 fs, 10 µJ, 9epi

PDB reference: 52.7 fs, 100 µJ, 9eph

Generation and measurement of FEL pulse profiles; supplementary figures and tables. DOI: 10.1107/S2052252525002660/car5003sup1.pdf

## Data Availability

Coordinates and structure factors for structures were deposited in the Protein Data Bank as indicated in Supplementary Tables S1 and S2. Additional data files used for the generation of *RIDL* maps are available upon request.
