# Supplementary material for: Damage before destruction? X-ray-induced changes in single-pulse serial femtosecond crystallography
Source: IUCrJ. 2025 Apr 14;12(Pt 3):358–71. doi: 10.1107/S2052252525002660 (PMC12044858; doi:10.1107/S2052252525002660)
Supplement: Supplementary file 1 [file m-12-00358-sup1.pdf]

IUCrJ

Volume 12 (2025)

Supporting information for article:

Damage before destruction? X-ray-induced changes in single-pulse serial femtosecond crystallography

Lewis J. Williams, Amy J. Thompson, Philipp Dijkstal, Martin Appleby, Greta Assman, Florian S. N. Dworkowski, Nicole Hiller, Chia-Ying Huang, Tom Mason, Samuel Perrett, Eduard Prat, Didier Voulot, Bill Pedrini, John H. Beale, Michael A. Hough, Jonathan A. R. Worrall and Robin L. Owen

S1. Generation and measurement of FEL pulse profiles

Four XFEL pulse durations were prepared with a constraint of maintaining an average FEL pulse energy of at least 200 μJ at the X-ray gas monitor (shown in figure 1a), to ensure that at least 100 μJ reached the sample position in the Cristallina experimental station, where the photon beam can optionally be attenuated. The shortest target value was 10 fs (configuration A). Further targets were 25 fs (B), the maximum possible pulse duration given the constraint on the pulse energy (D), and finally one intermediate value between the previous two targets (C). For A and B we employed a short electron bunch duration, and for A we additionally employed a beam shaping wakefield device. For C and D we consecutively increased the electron bunch duration.

We produced and measured three different electron bunch durations for the experiment, here designated as Short (for X-ray pulse durations of 8 fs and 22 fs), Medium (45 fs), and Long (55 fs) to generate the four pulse durations. The Medium and Long current profiles were measured twice, once in advance of the experiment on a beam preparation day and again immediately prior to the SFX measurements. The current profile measurements measured on different days show excellent agreement, indicating a stable bunch compression setup throughout several days of photon delivery. The FWHM pulse durations were 15 fs, 24 fs, and 34 fs, with a shot-to-shot jitter of ~5%. From the transverse deflecting structure (TDS) time calibration constant we expect a systematic error also at the 5% level. The durations obtained from the two different streaking directions in a TDS are different by less than 1%. From each measurement, we show in figure S1 the single shot current profile that has the on average smallest deviation compared to all other 24 measurements.

Figure 1d shows the FEL pulse measurements. The pulse duration τ extracted from a direct FWHM analysis provides unstable results in the case where the power profile has at least one broad flank. Therefore we instead performed an rms analysis and applied the factor 2.355, which relates rms and FWHM of a Gaussian pulse. Differences in duration between multiple measurements for the same machine configuration are between 4 and 10%. The 5% systematic error in the TDS-based calibration of the wakefield structure translates to a 5% error in τ. The wakefield structure has an rms time resolution r_t on the few fs level [6, 15], which causes an overestimation of τ. This is however only relevant for the shortest FEL pulses shown here. Assuming r_t = 2.5 fs, the true τ can be estimated as 7 fs instead of 9 fs.

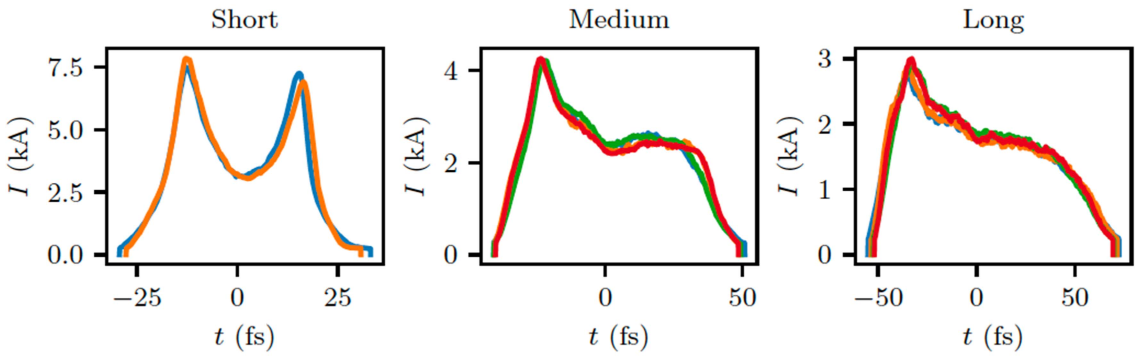

Figure S1 Measured electron beam current profiles recording using a transverse deflecting structure, showing results from both RF phase zero-crossings for the three electron bunch durations used.

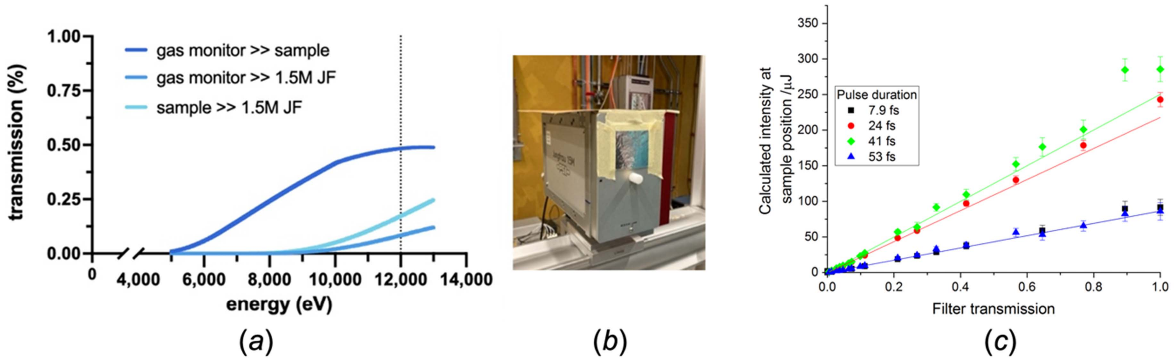

Figure S2 (*a*) plot showing the relative transmission at different beam energies of the beamline from the gas-monitor to the 1.5M Jungfrau used for the pulse energy calibration. The gas monitor gives an accurate readout of the shot-to-shot energy at the source(Tiedtke *et al.*, 2008, Juranic *et al.*, 2018). (*b*) The 1.5 M JF detector used for the direct beam photon energy calculations. To record the direct beam without any upstream attenuation, the detector cover (2 mm Al) with an additional 0.8 mm aluminium foil layer was used. (*c*) Calibration curves for each pulse duration of the back-calculated pulse energy at the sample position.

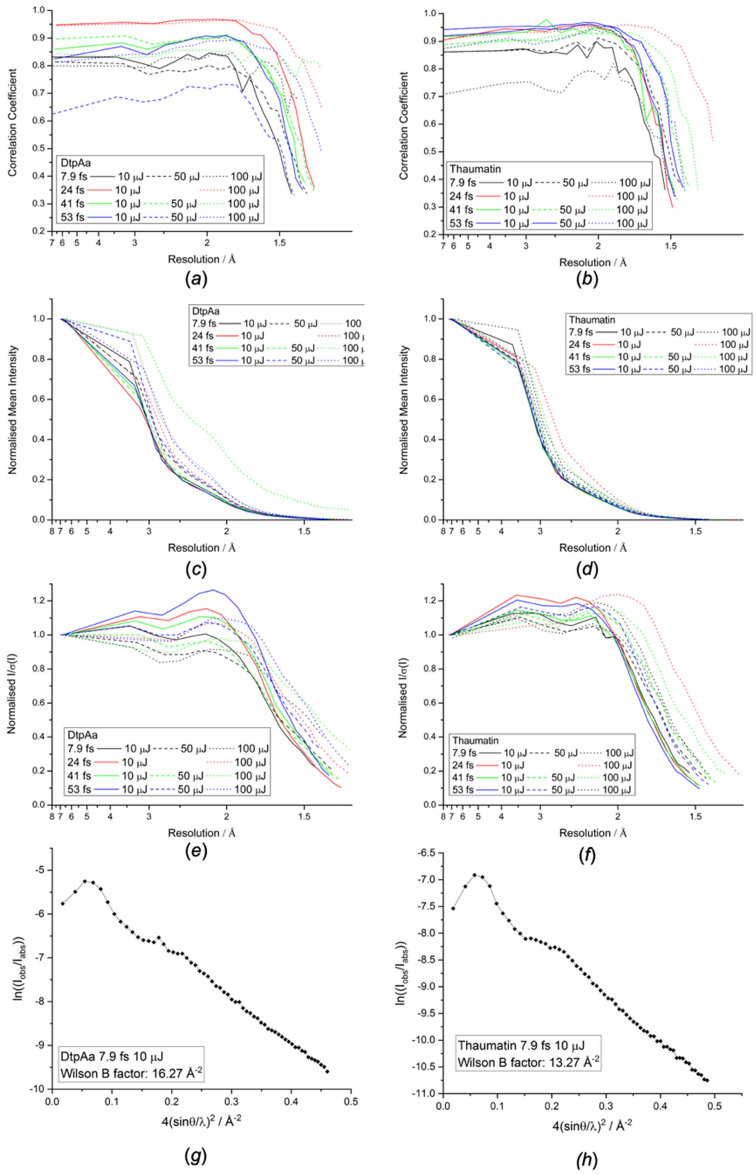
Correlation Coefficient
Resolution / Å
DtpAa
Thaumatin
7.9 fs 10 μJ 50 μJ 100 μJ
24 fs 10 μJ 100 μJ
41 fs 10 μJ 50 μJ 100 μJ
53 fs 10 μJ 50 μJ 100 μJ
(a)
(b)
Normalised Mean Intensity
(c)
(d)
Normalised I/σ(I)
(e)
(f)
ln((I_obs/I_abs))
4(sinθ/λ)^2 / Å^-2
DtpAa 7.9 fs 10 μJ
Wilson B factor: 16.27 Å^-2
Thaumatin 7.9 fs 10 μJ
Wilson B factor: 13.27 Å^-2
(g)
(h)

Figure S3 Data quality metrics for DtpAa and thaumatin as a function of resolution for all pulse duration and intensities. Correlation coefficient of DtpAa (*a*) and thaumatin (*b*), mean diffracted intensity of DtpAa (*c*) and thaumatin (*d*), and Mean I/σ(I) of DtpAa (*e*) and thaumatin (*f*). All statistics are as reported by CrystFEL. Panels (*g*) and (*h*) show exemplar Wilson plots for DtpAa and thaumatin 7.9 fs 10 μJ datasets as reported by Truncate.

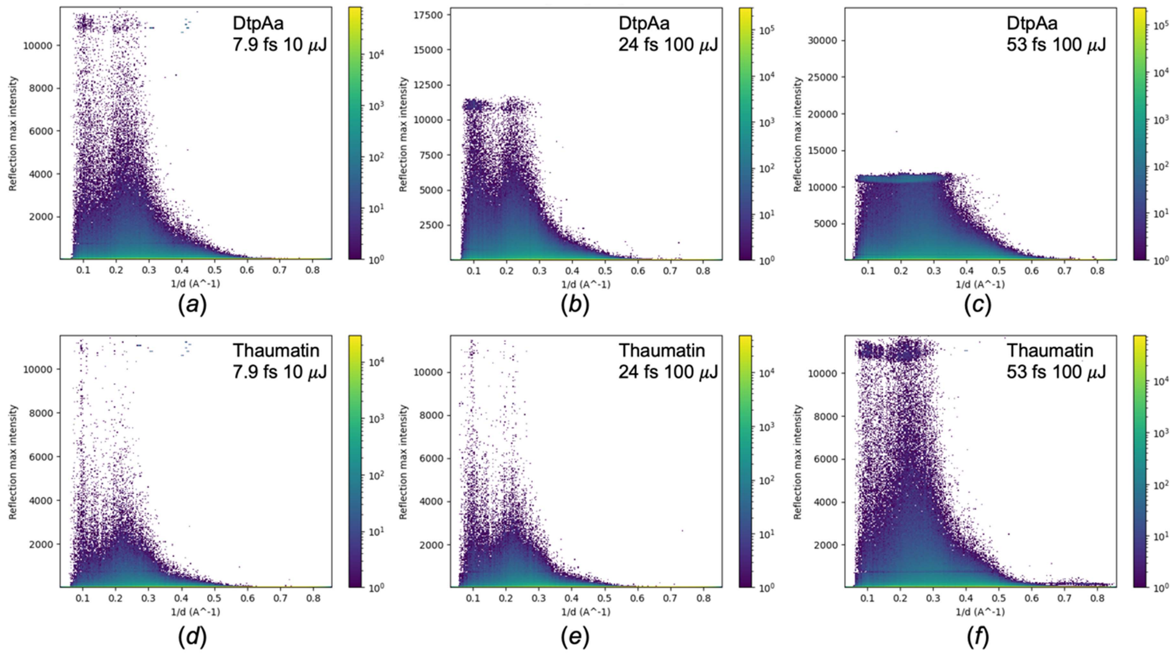

Figure S4 Detector saturation plots for the datasets: DtpAa 7.9 fs 10 μJ (*a*), DtpAa 24 fs 100 μJ (*b*), DtpAa 53 fs 100 μJ (*c*), thaumatin 7.9 fs 10 μJ (*d*), thaumatin 24 fs 10 μJ (*e*) and thaumatin 53 fs 100 μJ (*f*). A horizontal 'cloud' at the top of the maximum peak energy distributions indicates where detector saturation is occurring. More saturation is evident for the more strongly diffracting crystals of DtpAa than for thaumatin.

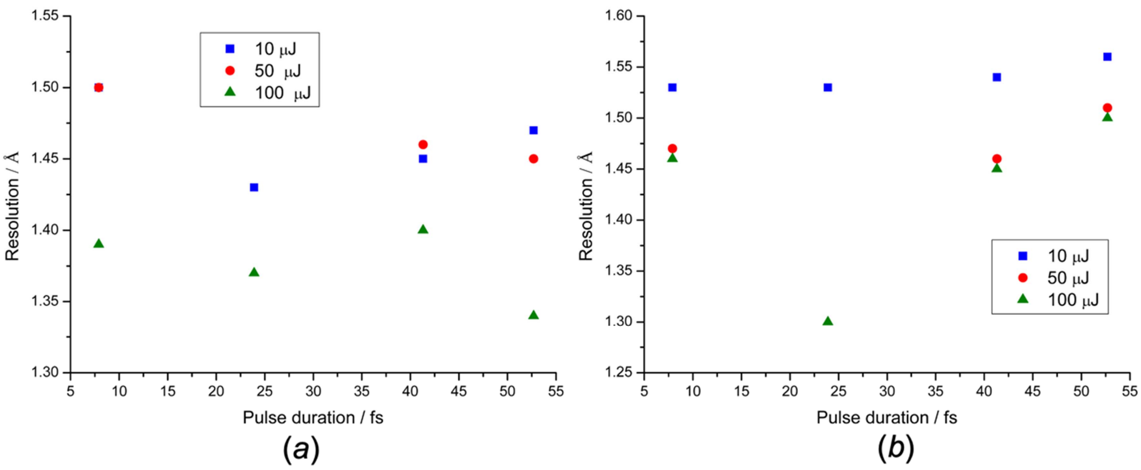

Figure S5

Comparison of dataset high resolution limit as determined by monotonic decay of CC to 0.3 for DtpAa (a) and thaumatin (b) as a function of pulse duration for 10 μJ (blue), 50 μJ (red) and 100 μJ (green) data. In all cases a subset of 6,200 images was used to determine the resolution cutoff in a consistent manner.

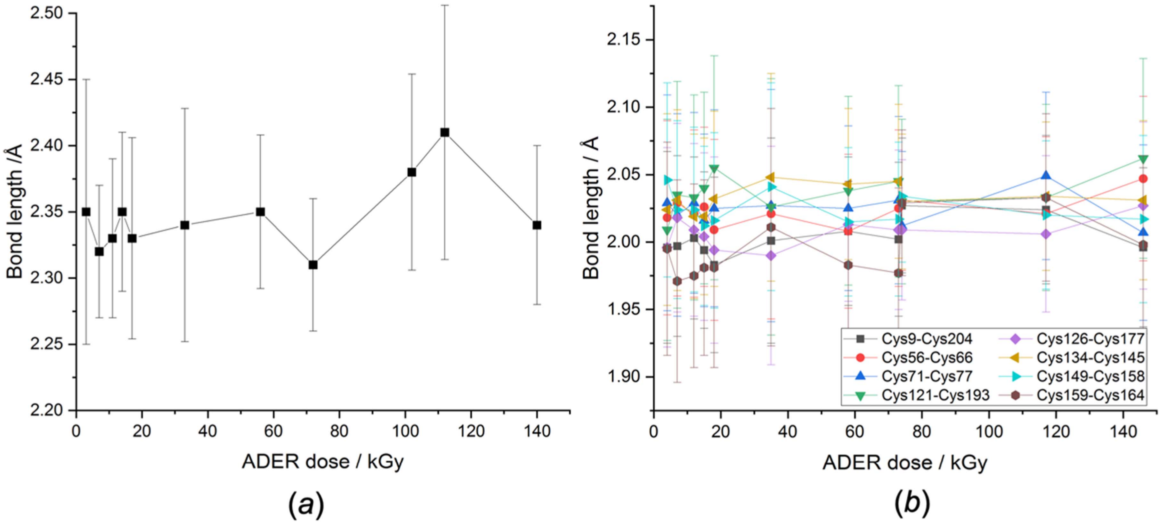

Figure S6 Variation in refined bond lengths as a function of ADER dose. Length of Fe(III)-H_2O bond length in DtpAa (*a*), and length all disulphide bonds in thaumatin (*b*).

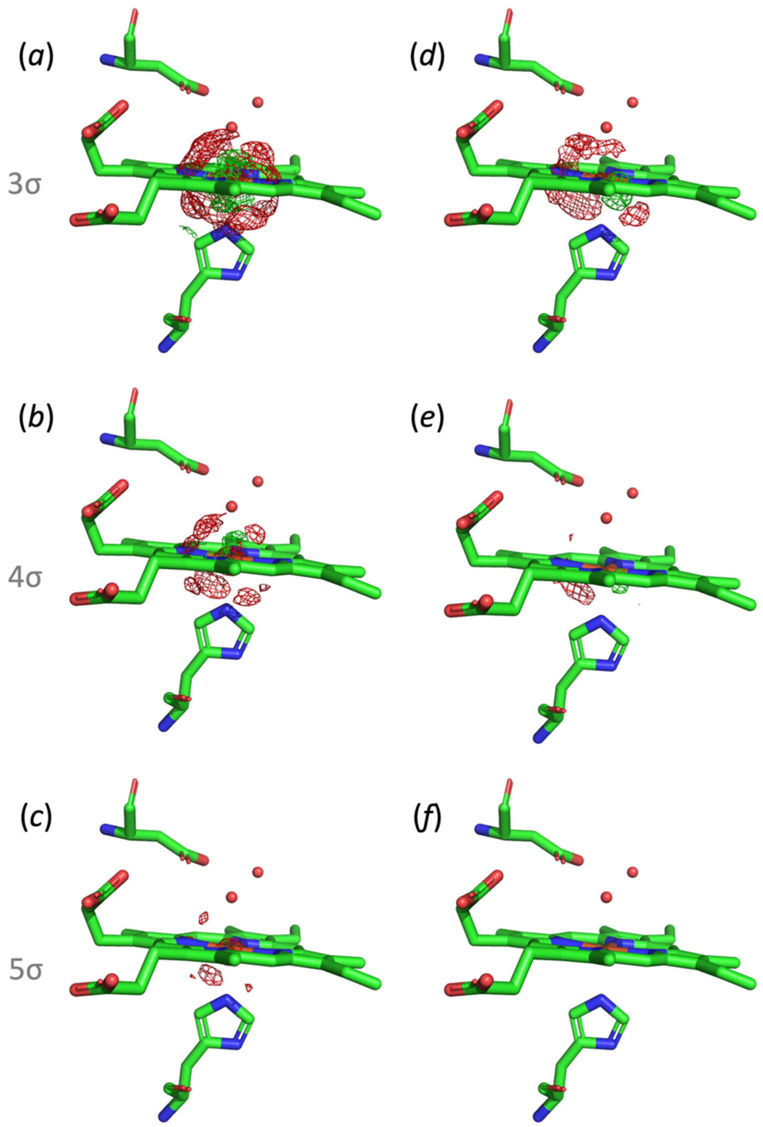

Figure S7 RIDL difference density maps of DtpAa between the 7.9 fs 10μJ and 7.9 fs 100 μJ datasets obtained without using consistent resolution cut-off (*a*) and with a global resolution cut-off of 1.75Å (*b*). As expected, imposition of a consistent resolution cut-off greatly reduces the level of Fourier truncation ripple features in the maps but interpretation can still challenging in the region surrounding the Fe atom and care must be taken as artefacts can remain that may not obviously 'ripples' (as seen for example in panel e).

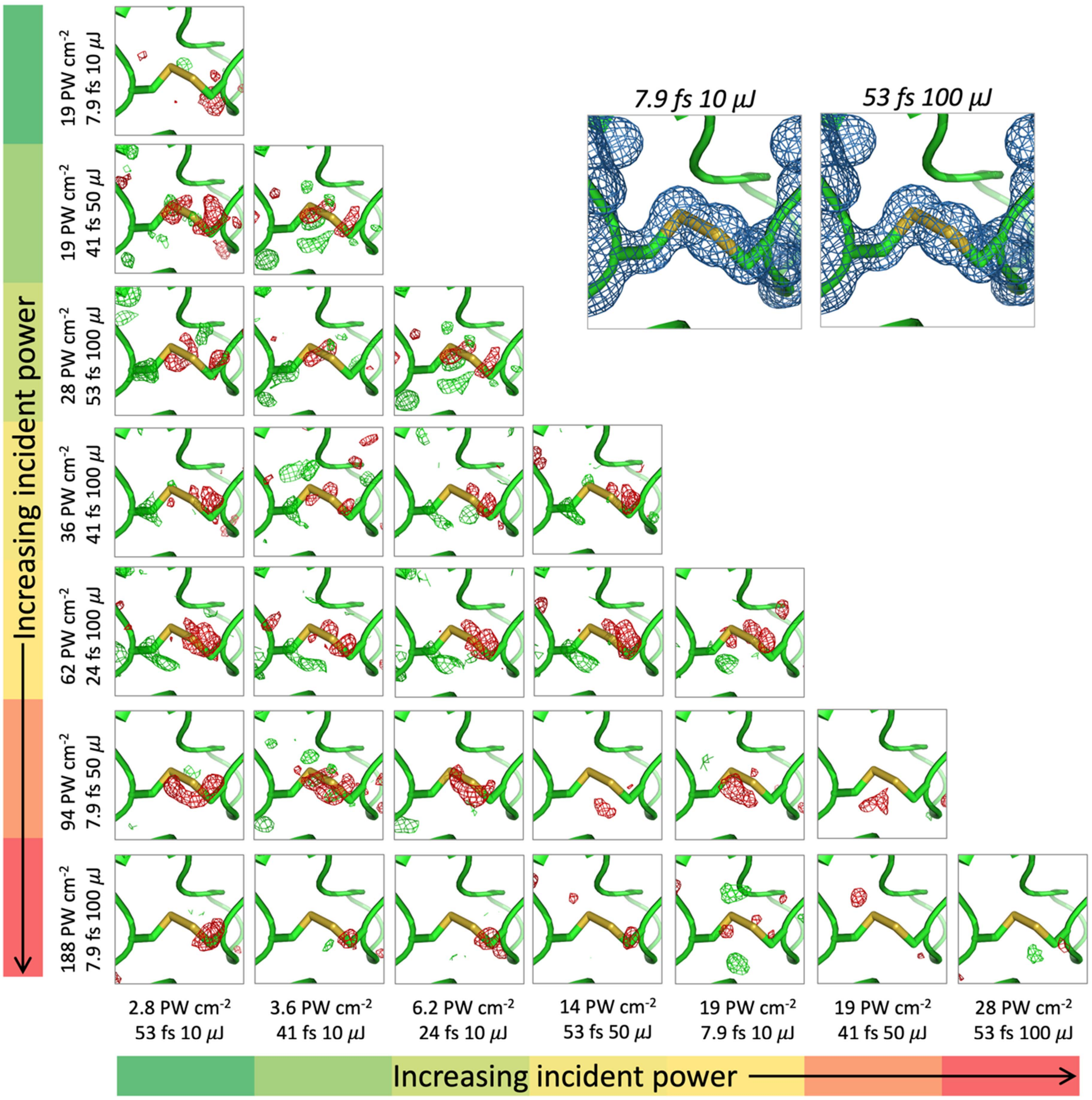

Figure S8 RIDL isomorphous electron density difference maps comparing the thaumatin datasets at the exemplar disulphide bond Cys134-Cys145. Rows are sorted in order of beam power with lowest powers at the top. All difference maps are contoured at 3σ. The inset shows 2Fo-Fc maps contoured at 1.5σ for 9 fs 10 μJ and 46 fs 100μJ data over the same region illustrating that these maps are essentially identical.

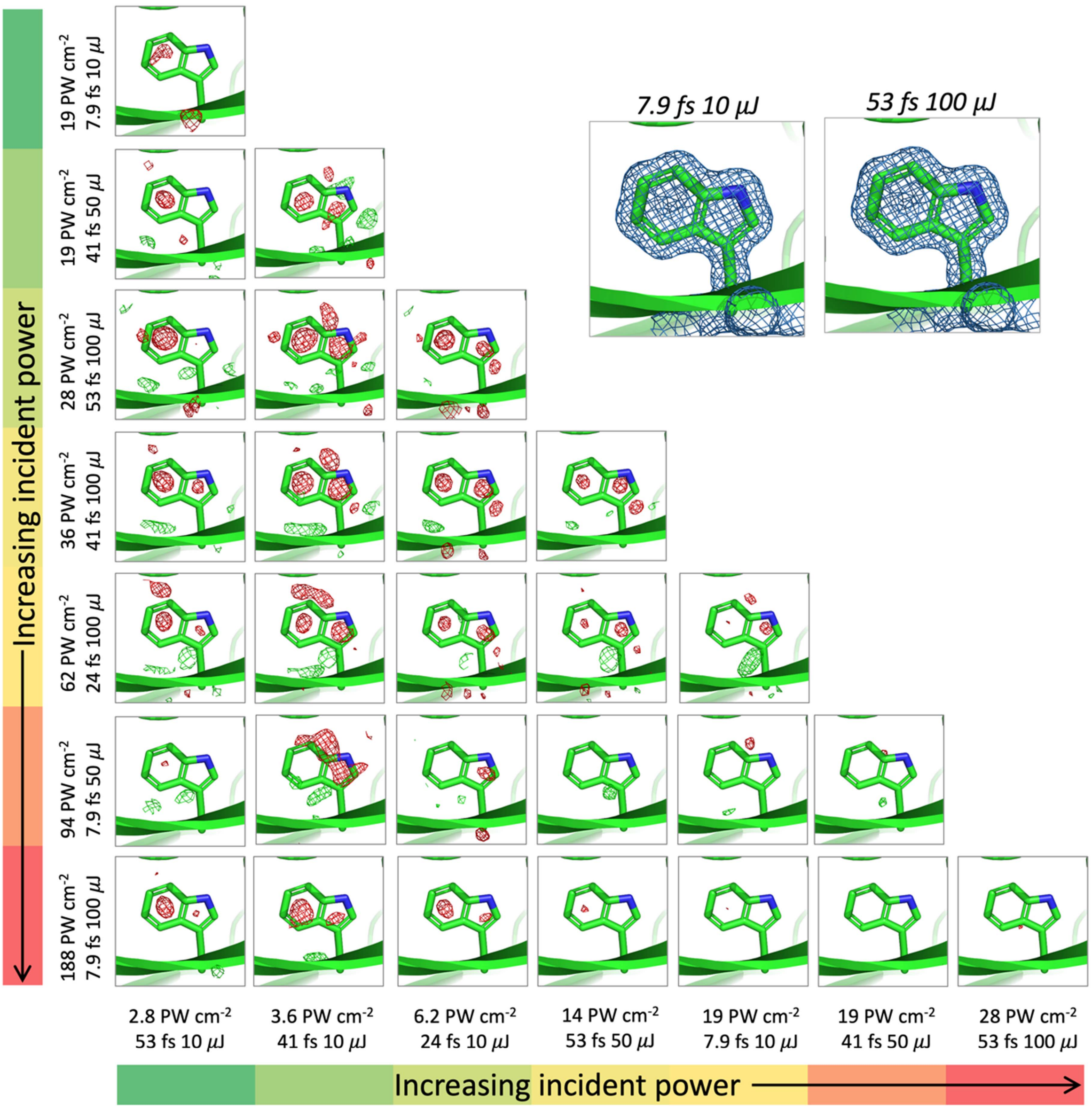

Figure S9 RIDL isomorphous difference electron density maps comparing the thaumatin datasets at tryptophan 51. Rows are sorted in order of beam power with lowest powers at the top. All difference maps are contoured at 3 σ. The inset shows 2Fo-Fc maps contoured at 1.5 σ for 9 fs 10 µJ and 55 fs 100µJ data over the same region illustrating that these maps are essentially identical.

Table S1 Scaling and refinement statistics for DtpAa datasets.

All DtpAa data were in space group $P2_1$ with a=72.7 Å, b=68.2 Å c=74.6Å Å and β=105.6°. ESU estimate based on Rfree set.

Dataset	7.9 fs 10 μJ	7.9 fs 50 μJ	7.9 fs 100 μJ	23.8 fs 10 μJ	23.8 fs 100 μJ	41.3 fs 10 μJ	41.3 fs 50 μJ	41.3 fs 100 μJ	52.7 fs 10 μJ	52.7 fs 50 μJ	52.7 fs 100 μJ
Resolution / Å	34.10 – 1.44 (1.47 – 1.44)	34.10 – 1.37 (1.40 – 1.37)	35.02 – 1.37 (1.40 – 1.37)	35.02 – 1.34 (1.37 – 1.34)	35.02 – 1.25 (1.27 – 1.25)	34.10 – 1.38 (1.41 – 1.38)	34.10 – 1.34 (1.37 – 1.34)	35.46 – 1.21 (1.23 – 1.21)	34.10 – 1.39 (1.42 – 1.39)	34.10 – 1.43 (1.46 – 1.43)	34.10 – 1.27 (1.29 – 1.27)
Merged crystals	11021	13534	17595	40030	57429	18275	21358	37427	14617	5997	21361
Unique reflections	129619 (6442)	150621 (7548)	150622 (7548)	160910 (7966)	198421 (9901)	147332 (7322)	160910 (7966)	218812 (10883)	144168 (7214)	132374 (6570)	189137 (9387)
Completeness (%)	100 (100)	100 (100)	100 (100)	100 (100)	100 (100)	100 (100)	100 (100)	100 (100)	100 (100)	100(99.98)	100 (100)
Multiplicity	71.3 (47.7)	136.4 (88.7)	209.5 (136.2)	247.0 (159.3)	483.9 (173.5)	115.7 (76.2)	215.8 (139.4)	246.8 (51.5)	76.0 (50.6)	59.0 (39.7)	179.3 (85.5)
$CC_{1/2}$	0.888 (0.336)	0.885 (0.337)	0.896 (0.487)	0.971 (0.360)	0.975 (0.316)	0.917 (0.376)	0.941 (0.347)	0.907 (0.709)	0.900 (0.351)	0.776 (0.332)	0.915 (0.315)
R_{split}	0.289 (1.101)	0.299 (1.130)	0.257 (0.631)	0.138 (1.069)	0.129 (1.262)	0.231 (1.036)	0.212 (1.152)	0.252 (1.082)	0.249 (1.175)	0.406 (1.061)	0.232 (1.251)
I/σ(I)	3.23 (1.01)	3.02 (0.98)	3.96 (1.79)	5.89 (0.95)	5.88 (0.83)	4.05 (1.02)	3.84 (0.94)	3.99 (0.96)	3.83 (0.85)	2.61 (1.11)	3.848 (0.82)
Wilson B / $Å^{-2}$	16.3	15.3	14.7	17.0	15.6	16.9	14.9	4.5	18.8	16.9	15.8
R_{work}	0.177			0.153	0.161	0.166			0.170		0.174

R_{free}	0.205	0.169	0.178	0.187	0.194	0.203
RMSD bond length (Å)	0.0112	0.0123	0.0124	0.0118	0.0112	0.0096
RMSD bond angles (°)	1.877	1.880	1.840	1.856	1.813	1.672
Ramachandran favoured (%)	97.22	97.09	97.08	97.22	96.94	97.36
ESU (Å)	0.073	0.046	0.046	0.058	0.058	0.046
PDB code	9EPD	9EPG	9EPK	9EPJ	9EPI	9EPH

Table S2 Scaling and refinement statistics for thaumatin datasets.

All thaumatin data were in space group $P4_12_12$ with a=b=58.5Å, c=151.3 Å. ESU estimate based on Rfree set

Dataset	7.9 fs 10 μJ	7.9 fs 50 μJ	7.9 fs 100 μJ	23.8 fs 10 μJ	23.8 fs 100 μJ	41.3 fs 10 μJ	41.3 fs 50 μJ	41.3 fs 100 μJ	52.7 fs 10 μJ	52.7 fs 50μJ	52.7 fs 100 μJ
Resolution / Å	31.98 – 1.53 (1.56 – 1.53)	31.98 – 1.45 (1.48 – 1.45)	31.98 – 1.47 (1.50 – 1.47)	31.98 – 1.49 (1.52 – 1.49)	31.98 – 1.27 (1.29 – 1.27)	31.98 – 1.48 (1.51 – 1.48)	31.98 – 1.41 (1.44 – 1.41)	31.98 – 1.37 (1.40 – 1.37)	31.98 – 1.48 (1.51 – 1.48)	31.98 – 1.44 (1.47 – 1.44)	31.98 – 1.43 (1.46 – 1.43)
Merged crystals	8240	12599	5716	12305	17473	13546	14756	14706	16593	15947	11780
Unique reflections	41507 (2043)	48619 (2383)	46664 (2257)	44873 (2222)	71976 (3542)	45753 (2221)	52804 (2594)	57471 (2785)	45753 (2221)	49611 (2416)	50650 (2460)
Completeness (%)	100 (100)	100 (100)	100 (100)	100 (100)	100 (100)	100 (100)	100 (100)	100 (100)	100 (100)	100 (100)	100 (100)
Multiplicity	70.8 (46.0)	129.8 (88.4)	69.2 (46.5)	116.1 (76.7)	295.1 (133.6)	122.2 (81.3)	166.2 (114.4)	202.8 (133.5)	148.9 (99.4)	195.7 (133.6)	161.0 (109.6)
$CC_{1/2}$	0.909 (0.362)	0.914 (0.385)	0.832 (0.329)	0.948 (0.300)	0.963 (0.353)	0.950 (0.357)	0.936 (0.370)	0.942 (0.360)	0.965 (0.339)	0.954 (0.370)	0.935 (0.359)
R_{split}	0.250 (1.129)	0.235 (1.274)	0.348 (1.280)	0.173 (1.356)	0.154 (1.328)	0.181 (1.405)	0.202 (1.246)	0.192 (0.970)	0.154 (1.438)	0.167 (1.245)	0.204 (1.142)
I/σ(I)	3.41 (0.88)	3.54 (0.81)	2.58 (0.84)	4.65 (0.77)	5.07 (0.73)	4.26 (0.71)	4.11 (0.78)	4.36 (1.10)	5.11 (0.72)	4.98 (0.85)	4.34 (0.94)
Wilson B / $Å^{-2}$	19.0	16.8	16.2	17.8	17.8	17.8	17.0	16.5	17.0	17.0	16.7
R_{work}	0.177	0.188	0.212	0.177	0.191	0.167	0.172	0.186	0.162	0.164	0.176
R_{free}	0.205	0.208	0.243	0.207	0.209	0.201	0.199	0.208	0.195	0.195	0.205
RMSD bond length (Å)	0.0092	0.0103	0.0085	0.0103	0.005	0.010	0.011	0.011	0.011	0.012	0.012
RMSD bond angles (°)	1.673	1.739	1.597	1.753	0.855	1.723	1.803	1.783	1.747	1.825	1.781
Ramachandran favoured (%)	95.34	97.40	95.88	96.37	98.5	96.35	96.35	96.88	96.35	97.40	96.35
ESU (Å)	0.068	0.060	0.072	0.063	0.052	0.063	0.054	0.052	0.062	0.054	0.057
PDB code	9EPE	9EQR	9EQS	9EQT	9EQU	9EQV	9EQX	9EQY	9EQZ	9ER0	9ER1

Table S3 . Dose calculations. RADDOSE-3D style average dose whole crystal (RD3D) and RADDOSE-XFEL average absorbed doses in the exposed region (ADER) in kGy for all datasets as calculated using RADDOSE-XFEL(Dickerson *et al.*, 2020).

Pulse duration / fs	7.9 ± 1.4			23.8 ± 0.6		41.3 ± 0.7			52.7 ± 2.5		
Pulse energy /μJ	10	50	100	10	100	10	50	100	10	50	100
Power density / ×10^{15} W cm^{-2}	19	94	188	6.2	62	3.6	18	36	2.8	14	28
DtpAa RD3D	21	104	209	21	206	20	102	204	21	102	205
DtpAa ADER	3	17	33	7	72	11	56	112	14	70	140
Thaumatin RD3D	23	115	230	23	232	23	117	235	24	118	236
Thaumatin ADER	4	18	35	7	74	12	58	117	15	73	146

Table S4 Heme A Fe(III)-H_20 bond lengths and variation in real space *B*-factors in $Å^2$ between DtpAa datasets

DtpAa structure	ADER dose (kGy)	Resolution (Å)	Heme A Fe(III)-H_2O (Å)	Chain A *B*-factor	Chain B *B*-factor	Average Protein *B*-Factor	Heme A *B*-factor	Heme B *B*-factor	Heme Average *B*-factor
7.9 fs 10 μJ	3	1.48	2.35 ± 0.10	18.7	17.3	18	11.1	11.2	11.15
23.8 fs 10 μJ	7	1.33	2.32 ± 0.05	20.2	19.3	19.75	13	13.3	13.15
23.8 fs 100 μJ	72	1.3	2.31 ± 0.05	19	18.1	18.55	12.1	12.6	12.35
41.3 fs 10 μJ	11	1.37	2.33 ± 0.06	19.5	18.4	18.95	12.2	12.5	12.35
52.7 fs 10 μJ	14	1.38	2.35 ± 0.06	19.8	19	19.4	12.5	12.9	12.7
52.7 fs 100 μJ	140	1.31	2.34 ± 0.06	19.1	18.1	18.6	12.2	12.8	12.5
